# A Memoir on the Yellow Fever of the West Indies, *as It Occurred in the Year* 1838, *at St. Pierre, Island of Martinique*

**Published:** 1840-01-25

**Authors:** E. Rufz

**Affiliations:** Adjunct Professor at the School of Medicine of Paris


### MEDICAL EXAMINER.

DEVOTED TO MEDICINE, SURGERY, AND THE COLLATERAL SCIENCES.

No. 4.] PHILADELPHIA, SATURDAY, JANUARY 25, 1840. [Vol. III.

### A MEMOIR ON THE YELLOW FEVER OF THE WEST INDIES,

*As it occurred in the year* 1838, *at St. Pierre, island of Martinique.* By E. Rufz, D.M.P., Adjunct Professor at the School of Medicine of Paris.

Translated for the Medical Examiner, from the French Manuscript.—[*Continued.*]

CASE IV.

M. Fraquet, a European, a year in the colony, thirty-two years old, of a strong constitution, was cured, soon after his arrival, of chancres and a venereal sore throat, which he had had during the whole passage. He was teacher in a school, and led a sober and regular, but sad and solitary life. He lived near the fort, in a high, well ventilated quarter, at a distance from the shipping.

On the 20th of October he had a violent headach, with fever and delirium at night. On the 21st he was bled. The symptoms continued; he was again bled; vomiting occurred on the fourth day; no dejections. On the fifth complete stupor; up to that time there was no remission, much less interruption in the progress of the symptoms.

On the 27th of October, seventh day of the disease, I was called in consultation by Dr. Noverre. There was much stupor; the patient scarcely understood our questions, but his answers were correct; pupils dilated; conjunctivæ injected; tongue red, moist, clear, tremulous, and protruded with difficulty; deglutition difficult. There was some tenderness on pressure at the region of the larynx; no return of vomiting; abdomen flaccid, and not tender; stools, only by the aid of injections; yellow colour of the whole skin and conjunctivæ; uniform heat; dorsal decubitus; the patient is able to sit up, and seems to retain his strength; pulse full and regular, eighty-four. Frictions, with Huxham's tincture and the sulphate of quinine; flying blisters the day before to the legs; two grains of sulphate of iron, with enema of powdered cinchona in Madeira wine.

I had seen the patient at 3 o'clock in the afternoon; the symptoms increased, and at 8 P. M., there were vomiting and stools of pure blood; death at midnight.

*Autopsy the* 28*th, at* 10 *A. M.*—The exterior was yellow, with a deep velvet tint in the dependent parts; no putrefaction.

The scalp and the sinus of the brain are full of black fluid blood. The vessels of the pia mater are also filled with the same blood. There is a slight sero-sanguinolent infiltration beneath the arachnoid, at the posterior and inferior part of the cerebral mass. Four spoonsful of serum in the ventricles. The arachnoid may be everywhere easily detached. The two cerebral substances are very firm and injected. The lungs are healthy, except some engorgement at their posterior part; the heart is equally normal. The pericardium and pleuræ contain no serum; the peritoneum is viscid to the touch, and much injected over the intestines. The stomach is dilated, and contains about a tumblerful of black and almost pure blood. It is throughout finely injected, with here and there dots like flea-bites. The mucous membrane is puckered; its consistence is generally good,—but in the great tuberosity it is more adherent, and breaks readily when raised up in strips.

The small intestines are rather contracted than dilated. Their mucous membrane is covered by a grayish substance, and presents here and there points like the stomach, of black blood. Some of these are patches, nearly half an inch broad; they are evidently sub-mucous ecchymoses, for the mucous coat is not altered. The coat itself is reddish and brittle, but not softened. The glands of Brunner are a little larger than usual, but the glands of Peyer are quite natural.

The large intestine is filled with an abundant and almost pure black blood; the mucous coat is reddish, but not altered. The spleen is large, but of good consistence. The kidneys are filled with blood; the bladder contains about three ounces of pure blood in its cavity. There are patches of ecchymosed blood, similar to those in the intestine, but its consistence is good. The liver is of ordinary size,—yellow, like that of fowls; its incised surface is gray. The large vessels are filled with black blood. The gall-bladder is filled by a thick, blackish bile; its mucous membrane is reddish, but of good consistence.

All the cellular tissue of the different organs is yellowish.

This is evidently a case of yellow fever, such as it is described. I regret that this history of the symptoms is not more complete, but this defect may be supplied by other cases which are more detailed. I will return again to the anatomical alterations in a particular chapter.

I have selected this case, because it is one of the first which I observed, and is characteristic of the disease. The cases increased. In November I saw seven, December fifteen,

January nine, February eleven, March five, April eight, May five, June six.*

*Mode of attack of the disease.*—The attack was often sudden. A person in good health might be seated at table, or engaged with business, and in two hours you would find him in a high fever. Or he would lie down in good health, and awake in the night with all the symptoms of the disease. (See cases.) It was less common for the attack to come on slowly; then the patients would feel, for several days before, a sensation of weight in the head, malaise, loss of appetite, with a whitish tongue, and a disagreeable characteristic breath. Sometimes there was a little cough. (See case of Mdlle. Eliza.) I have seen one or two with a real chill, so that one would think that the disease was beginning, and afterwards feel nothing for one or two days. The fever would then set in, and continue. The form would be almost intermittent at the beginning.

In more than three-fourths of the cases, the attack commences with a single regular chill, whether the disease comes on suddenly, or has been preceded by premonitory symptoms. The chill may be wanting, (see the case of Lucotte, 6th.)

This chill is not of long duration, and is followed by a lively heat, accompanied by a severe pain in the head, embracing, like a crown, the forehead and particularly the supra-orbitary arches; this pain in the head continues without intermission.

The face is red, approaching to violet; the conjunctivæ injected and shining; the pupil dilated; the eye bears with difficulty the impression of light.

The intellectual faculties are torpid; the patient does not even think of the most urgent business; he often shows great anxiety concerning the termination of the disease, or he continues to repeat that it is not the yellow fever.

The skin is warm, and often dry; but it is almost as often somewhat moist. Generally, after some hours, it is red over the whole body. The pulse is full and frequent, from one hundred to one hundred and twelve, but usually soft and easily compressed. The lumbar region and the extremities, particularly the lower, are the seat of pains which compel the patients to toss about continually. I saw one case in which the pains in the lower extremities were so insupportable as to require special treatment, (frictions and cataplasms.)†

The tongue is sometimes natural, but in a majority of cases it is whitish, approaching to yellow, and rather thickly coated; its point, moreover, is often red; it is rarely dry.

The lips are dry; the mouth clammy, with a disagreeable taste; the breath very disagreeable, with a characteristic odour. A disgust for drinks is more frequent than great thirst. Deglutition is unobstructed.

I have very rarely found the epigastrium painful to the touch, this region, as well as the whole abdomen, being almost always yielding and entirely free from pain. The arterial pulsations of the epigastrium to which M. Maker, (Annales Maritimes) following M. Belot of Havana, attaches great importance as a prognostic sign, did not present to me any peculiar character; they were, like the pulsations of the arteries generally, more or less marked, according to the greater or less intensity of the fever. There was often a sense of fulness in the stomach, with eructations, nausea, and sometimes one or two vomitings; but I have never seen in this epidemic, vomiting commence at the beginning and continue to the end without intermission, which is said to have occurred in other epidemics. The matter vomited was whitish or greenish.

The stools are rare; the urine scanty; the respiration a little accelerated. In three cases there was some cough, dry and very fatiguing; in one, this symptom was so marked, that one of the physicians called in consultation maintained that it was a case of pleurisy; but auscultation removed this doubt, for the respiration was every where very perceptible and unattended with râle.

This is a picture of the disease in its early stage.

*Progress of the disease—first period.*—These primary symptoms continued with the same intensity during twenty-four, forty-eight, and even sixty hours. There was no intermission, and the exacerbations were hardly perceptible. However the patients commonly said that they were at one time worse, at another time better, especially towards evening; but these exacerbations were neither appreciable by an augmentation of the pulse, nor by an increase of temperature; they were chiefly indicated by a greater degree of agitation.

The first night there was a heavy sleep, by no means refreshing, and frequently interrupted by harrassing dreams, or slight delirium. The following nights the insomnia was complete. The cephalalgia, even when it was not combatted by any treatment and when the disease was aggravated, did not last beyond the second or third day; it was replaced by a sense of weight in the head. Vomiting, when it occurred, appeared to diminish the cephalalgia;

* These are only the number of patients in my practice. In the hospital, the largest number of patients from the garrison was in the month of October, when there were two hundred and twenty-nine admissions; the smallest in December, when there were fifty-seven. In June, the last month of the series, there were sixty-seven.

† I have heard cited five or six cases in which these pains indicated large effusions of blood in the thickness of the limb, (see Velm) and consequently the formation of large abscesses when the disease was protracted.

but it yielded particularly to bleeding, sometimes to a marvellous degree. In proportion as the blood flowed, the patient experienced relief; but after an interval of some hours, the cephalalgia most commonly returned with as much severity as it exhibited before the bleeding. Cold applications to the head afforded some relief.

The pains in the loins and extremities pursued exactly the same course as the cephalalgia. When the disease did not terminate frankly, they were replaced by a sensation of general fatigue.

The heat became more and more dry, especially when the termination was not speedy.

The pulse continued to give the same number of pulsations, but it was often accelerated after bleeding, and became harder.

The injection of the eyes and face was diminished by venesection; that of the face, however, in many cases, continued after the most copious bleedings. The state of the pupils was variable, but in the greater number of cases they remained dilated.

The torpor of the intellectual faculties decreased, but there was always very great anxiety.

The tongue became more red at its tip. If there was thirst, it generally diminished ; but I saw one case in which it was irremediable during four days ; the nausea augmented.

Vomiting, I have said was rare during the first period ; the stools were also rare and the discharges of urine no less so ; most commonly, an evacuation could only be procured by an enema. The abdomen continued in the same state, yielding and free from pain.

The respiration was easy and free. In one of the four cases in which there was cough, it ceased after the bleeding, and in the other three it continued during the whole of this period.

In more than one-third of the cases which came under my observation, after the third day all the symptoms had lost some of their severity, the skin was more moist, the pulse almost natural and the urine easily passed. In one or two cases I saw epistaxis supervene at this time, and the patients exhibited signs of recovery: but generally these appearances were too slight and too rare too be considered as critical affections.

Speedily fatal termination.

In other cases the disease made a rapidly fatal progress. Some hours after its commencement, coma, delirium, icterus, suppression of urine, and black vomit succeeded, and death took place on the second day. Such is the case of Ventieghem ; it is the only case of the kind that I have observed during this epidemic; but I heard mentioned, and read in the works of Perguet and Dariste, of more than one similar death during the preceding epidemics, (1802 and 1816.)

CASE V.

Ventieghem, a Belgian, aged 38 years, of a robust constitution, has sailed for four or five years from Europe to the Antilles. In September, 1837, he was treated by me for an attack of sporadic cholera morbus. He was in great danger from this attack, and was only restored to health by returning to Europe.

He came back to Martinique in January, 1839 ; the epidemic was then at its height. Ventieghem, during the passage, had committed great excesses in the use of alcoholic liquors, and he did not refrain from them after his arrival, although he greatly dreaded the disease.

I saw him on the 5th of January, at eight o'clock in the morning. He was well, and merely perceived that his strength had diminished, and that he could not raise with the same exertion as formerly, the packages which he had been accustomed to lift.

Towards 10 o'clock, he felt an indefinable uneasiness, and had a feeling of weakness about him. At noon, I found him in the following state : face flushed, eyes injected, extremities cold, skin warm ; the pulse, however, soft and frequent; very great pain in the head; tongue whitish, moist and quite natural ; neither nausea nor vomiting ; no colic.

The chill lasted a half-hour, and after it was over, I took sixteen ounces of blood from him.

The patient at the conclusion of the bleeding, fainted and vomited some greenish matter. The blood was black and very warm ; one-third serum, and no buff.

Towards evening the cephalalgia was diminished ; there existed, however, during the whole time, a feeling of stricture at the frontal region. The face was flushed ; perspiration copious ; thirst excessive ; three stools after injections ; pulse soft enough and compressed. I directed fifty leeches behind the ears : these leeches drew little blood.

At six in the morning I found him in the following state : his face was of a purplish red, and swelled ; his eyes closed and injected; his ideas confused ; (he had had delirium in the night;) the cephalalgia continued ; his tongue was tremulous ; his thirst excessive ; his pulse soft and frequent; his abdomen slightly meteorized ; no evacuation from the bowels ; a complete suppression of urine since yesterday evening ; decubitus dorsal.

I took sixteen ounces of blood, the loss of which was followed by weakness, and a greenish evacuation. Sixty leeches were applied to the epigastrium.

At ten o'clock the bleeding was repeated to the amount of eight ounces, and was followed by a loss of strength.

Almost immediately after this third bleeding, the state of the patient grew worse every hour. The surface of the body assumed a yellowish colour ; at noon the patient had almost entirely lost his perception, and experienced great pain in swallowing.

In vain they put blisters on his extremities; and they tried to give him injections of quinine; nothing could rouse him, and in scarcely twenty-four hours after the commencement of the attack, he might have been considered moribund.

He died at eleven at night. Some moments before his death, a quack who was called in gave him an emetic, and he threw up a large quantity of black matter.

After death his body became of a greenish yellow colour. An autopsy was not made.

*Second period.*—But most commonly, the symptoms were not so rapid in their course. Towards the third day, when the termination was not decidedly favourable, there took place in the state of the patient, a perceptible change which authors have designated by the term, *period of repose.* In effect, the cephalalgia diminished very much or even disappeared entirely; the eyes were less injected; the face became of a darker red, which, when dissipated by the pressure of the finger, was more slowly restored. The tongue remained always coated along the middle, and red at the edges, as at the commencement of the disease; the thirst, when it had occurred, was much diminished.

The paroxysms of vomiting, if any had existed in the early stage, lost their frequency; or in some cases, it was at this time that the patient began to suffer with nausea and eructations, the precursors of this vomiting.

The epigastrium, and the abdomen generally, were almost always in their natural state. Once or twice I saw patients who complained of a feeling of fulness in the epigastrium, which made them wish to take an emetic.

Stools were obtained more easily by clysters; the matter evacuated was grayish or yellowish. In some very rare cases, two or three, in which there had been diarrhœa from the commencement, this diarrhœa ceased; or, if there had been none before, it began to establish itself.

The urine still flowed, but at greater intervals, and became more turbid.

The respiration was sometimes slow and sighing.

The skin was cooler, softer, and more minute; the pulse fell successively, from 112 to 92, 84 and 76. It was soft and regular, so that judging from these two last symptoms, the pulse and heat of skin, one would have pronounced the patient ready to enter into the convalescent stage.

But this calm was fallacious. It moreover was not in accordance with the agitated state of the patients, who turned in every direction on their beds. They often went from one bed to another, but these efforts were frequently followed by debility. There was a want of sleep, and a general uneasiness, which cannot be defined; their speech was abrupt; the patients were in an ill-humour; they were peevish and restless in the extreme, and their countenances becoming more dull, assumed a sinister appearance.

This false remission lasted after the third, and even half of the fourth day, twenty-four or thirty-six hours.

In nearly half the cases, the disease, having reached this point, began to abate, and then proceeded to a favourable termination.

It was at this period that the hæmorrhages commenced from the nose and mouth, when they did take place, (see the case of Lucotte.) Often, also, some blackish matter was vomited, resembling soot suspended in a glairy fluid.

When the termination was to be fatal, the face was distorted, and extreme anxiety was pictured in every feature. The eyelids remained half closed, the conjunctivæ became yellow, and in a short time the icterus was general. The tongue and lips were tremulous; the articulation abrupt and impeded.

Hæmorrhage from the nose and mouth came on now, or continued, if they had before taken place. The lips were covered with a very thick layer of black blood.

Vomiting occurred more frequently, and was renewed by the least movement on the part of the patient. The black matter was more abundant, and often mixed with some streaks of pure blood.

The urine was suppressed, or was very scanty, and of a dark brownish colour. The stools were grayish or bloody.

There was often added to these symptoms, considerable oppression. M. de L., one or two hours before his death, complained of an intolerable pain in the præcordial region.

The pulse remained until the last moments calm and deceptive. More frequently it diminished in frequency from 60 to 50, grew more feeble, and then became insensible. It was at this time that the coldness of the extremities came on.

Death rarely took place before the fourth or after the seventh day. The last period frequently did not last longer than one or two hours, or at the most, seven or eight. Some patients fell into a profoud coma, others retained their intellectual powers; some had convulsions, or a great degree of agitation, and even a little delirium.

I have not seen a single case of carphologia, and but two of subsultus tendinum. Once only I saw a petechial eruption supervene on the third or fourth day. I have never observed a swelling of the parotid glands of any of my patients; and I have ascertained that among the great numbers admitted into the hospital, this symptom was observed but twice. Only once was a gangrenous slough with swelling of a limb seen there. This slough was probably an abscess following one of those effusions of which I have spoken. But blisters very frequently produced sloughs.

Icterus existed in all those cases which terminated fatally; it became very manifest after death, when it had not been perceived during life. I observed this symptom but twice among those who recovered. We see, then, that, although this symptom has served to give to the disease the name of yellow fever, it is far from being so frequent as might be supposed. Of 323 cases at Havana, M. Maker observed icterus in thirty-six only. The prognosis derived from the appearance of this symptom is rarely fallacious. If icterus appears before the seventh day, the case is almost always a fatal one: after the seventh, its appearance is a favourable symptom.

It is the same case with the black vomit, which hardly ever exists, except in cases of the gravest character; I have always seen it announce death. In the 323 cases above mentioned, M. Maker observed it but seven times. In all the cases which I saw, one only excepted, it was followed by death. Therefore, it should be with yellow fever as it is with variola; there should be *yellow fever without jaundice*, and *black vomit* without *black vomiting*, as there has been described *variola sine variolis.*

As for hæmorrhages, that from the intestines is less grave than that from the stomach. The case of Mad. Eliza offers a remarkable example of this (see case.) Epistaxis is less grave than hæmorrhage from the mouth, and the latter is rather favourable than otherwise, in the opinion of M. Catel, who observed it very often in the hospital. *During the course of the present epidemic*, I have neither seen nor heard of any case of hæmorrhage from the conjunctiva, the meatus auditorius, the skin, blisters, or the puncture made in venesection, examples of which are reported to have occurred during the other epidemics. In general, this hæmorrhagic tendency has appeared to me to be a phenomenon of less importance, than I had been led to consider it, by reading authors on this subject.

These hæmorrhages were frequent in the months of November and December, and subsequently in May and June; that is, at the commencement and towards the end of the epidemic. In the intermediate months this was a rare symptom. All the physicians of the city have been struck with this observation.

The blood drawn from the vein was very warm, and of a very dark colour, but soon became red by exposure to the air: it never presented a buffy appearance. The coagulum was very thick, and the serum by no means abundant.

Suppression of urine should cause very great apprehension, but it was not always followed by death. Bloody stools were often preceded by those of a grayish colour; there was generally constipation. The case in which diarrhœa occurred on the fourth day, terminated fatally.

I shall not speak of the prognostic value of other signs. In general, it may be said that there are few diseases the prognosis of which is better established, for it has been well studied in this respect.

I applied myself to the task of finding out if, at the commencement of the attack, it were possible to obtain from the circumstances connected with the case, any evidence as to the gravity of the disease. I found but four circumstances which appeared to me worthy of consideration,—1st. The constitution of the patient. 2d. His earlier or later arrival in the country. 3d. His manner of life. 4th. The period of the attack in which he commenced to be treated. As these four circumstances are, so to say, extraneous to the disease, I shall speak of them hereafter in the chapters relating to them. As for the disease itself, I found nothing in the early symptoms which could give any clue as to its termination. A severe chill was sometimes followed by a slight sickness; the other cases commencing without a chill terminated unfavourably. The violence of the febrile action, and the pains in the loins, were not always proportionate to the succeeding state of the disease. M. Maker, in his memoir, insists much on the pulsations of the cœliac artery in the epigastrium, as a prognostic sign; but the cœliac artery beats like all other arteries. This sign is moreover very difficult to appreciate.

[*To be continued.*]

